# Memory box: possibilities to support grief in the intensive care unit during the COVID-19 pandemic

**DOI:** 10.5935/0103-507X.20200079

**Published:** 2020

**Authors:** Thábata da Silva Cardoso Luiz, Orli Carvalho da Silva Filho, Tânia Cristina Camacho Ventura, Virginia Dresch

**Affiliations:** 1 Hospital Universitário Antônio Pedro, Universidade Federal Fluminense - Niterói (RJ), Brazil.; 2 Instituto Nacional da Saúde da Mulher, da Criança e do Adolescente Fernandes Figueira, Fundação Oswaldo Cruz - Rio de Janeiro (RJ), Brazil.; 3 Institute of Psychology, Universidade Federal Fluminense - Niterói (RJ), Brazil.

Dear Editor,

Grief is a process of individual adaptation, and a universal experience marked by subjectivity and culture. Most individuals cope with the reality of loss. However, when there is a desire for, or a persistent concern with the deceased, accompanied by intense emotional suffering and functional impairment for at least 6 months, it is called prolonged grief disorder (PGD).^(1)^ The prevalence of PGD varies according to the groups studied: from 10% in community samples exposed to non-violent death to 52% in family members of patients in the intensive care unit (ICU).^(2,3)^

In the current pandemic, countless families have suffered significant losses. Some variables that complicate the grieving process may be identified: communication difficulty, unexpected deaths, distance from the socio-affective network, the impossibility of bidding farewell, and prohibition of funeral traditions.^(4)^ These situations experienced in ICUs may impact the experience of terminality and predict risks to the mental health of relatives and healthcare professionals.

Therefore, we should understand that the Coronavirus 2019 disease (COVID-19) amplifies the challenges in the face of death, and to think about emotional support for the bereaved is urgent. We wrote this letter to stimulate the (re)creation of compassionate actions, to allow a better experience of care in the ICUs. It is fundamental for the grieving process to think of strategies to reconfigure the symbolic rituals, in which the bereaved can validate their relationship with their loved ones.^(5)^ Thus, we report an intervention developed by the mental health team of a university hospital, that sought to resignify the handling of death: the Memory Box.

This university hospital is a high-complexity unit in the metropolitan region of the capital of the State of Rio de Janeiro treating COVID-19 patients. In the course of the pandemic, the outburst of an intensivist drew attention to a practice adopted in compliance with biosafety standards. After communicating the death and delivering the patient’s belongings in a “disposal bag” to the family, he reported extreme anguish: “it was like life was no longer there, but the disease instead!”

From this experience, the mental health team proposed a change in the protocols for handing over the deceased patients’ belongings to their families, using the symbolic resource Memory Box. The Box is delicate, decorated with flowers, and contains some of the patient’s objects in the process of decontamination; it is accompanied by a message inviting to honor the life of the deceased by building good memories. It is delivered by the team to the relatives during a respectful conversation, affectionately encouraging them to keep the precious and special things belonging to that bond because affective memories can remain protected. The Memory Box makes concrete the recognition that good moments were shared: a gift from life. Therefore, death does not end this relationship, and the Box provides a worthy way to return home.

In the period from May 25, 2020, to July 3, 2020, 23 boxes were delivered to bereaved relatives. The acknowledgment of this action by the relatives is a constant, as well as the engagement of the different professionals involved, who describe relief and hope by introducing a comforting element into such a painful time. After the delivery, some family members request psychological accompaniment with the team.

Although systematization is required to appropriately assess this intervention on PGD during and after the pandemic, public health must invest and disseminate accessible, replicable, and low-cost early interventions.

As well as we seek to flatten the curve of severe acute respiratory syndrome coronavirus 2 (Sars-Cov-2) contamination, anticipating the mourning curve complicated by clinical teams is urgent. The integration of mental health actions in the ICU is a necessary, and still challenging reality.^(6)^ Providing bereavement support is one of the possibilities we consider when fighting this pandemic perspective.
